# Comprehensive investigation on flavonoids metabolites of Longjing tea in different cultivars, geographical origins, and storage time

**DOI:** 10.1016/j.heliyon.2023.e17305

**Published:** 2023-06-14

**Authors:** Xiao-Lan Yu, Jia Li, Yanqing Yang, Jiayi Zhu, Haibo Yuan, Yongwen Jiang

**Affiliations:** Tea Research Institute, Chinese Academy of Agricultural Sciences, 9 Meiling South Road, Hangzhou, Zhejiang 310008, PR China

**Keywords:** Cultivar, Glycosidification, Methylation, Chalcone, Flavonoid marker

## Abstract

In this study, four kinds of Longjing tea, the famous flat green tea and the protected geographical indication product in China, were used to explore the quality difference of the same green tea due to the cultivar, geographic origin, and storage time under the premise of consistent picking conditions and processing technology using the widely targeted metabolomics. Results showed that 483 flavonoid metabolites with 10 subgroups of flavonoids were screened and 118 differential flavonoid metabolites were identified. The number and subgroups of differential flavonoid metabolites produced by different cultivars of Longjing tea were the largest, followed by storage time, and third by the geographic origin. Glycosidification and methylation or methoxylation were the main structural modifications of differential flavonoid metabolites. This study has enriched the understanding of the effects of the cultivar, the geographic origin, and the storage time on the flavonoid metabolic profiles of Longjing tea, and provided worthy information for the traceability of green tea.

## 1 Introduction

Green tea, the unfermented tea made from fresh tea leaves of the *Camellia sinensis* plant, bearing its unique flavor [1,2] and health benefits [3,4], is being loved and consumed throughout all over the world; then comes the growing concern about the authenticity of green tea. Tea authenticity has different aspects [5], for example, the cultivar of the tea plant, the geographic origin, the type of tea, and the age of tea. In green tea, the cultivar, the geographic origin, and the age (the storage time) are the major aspects of tea authenticity. Taking the famous green tea, Longjing tea, the protected geographical indication product in China with its features of emerald green color, beautiful shape, rich fragrance, and mellow taste, for instance, the cultivars and the graded protection area (seen Fig. S1) are detailed stipulated in the national standard [6]. It is common knowledge that the cultivar, the geographic origin, the harvest time, and the edaphoclimatic condition can greatly influence the concentration and composition of bioactive chemical constituents in natural products [[7], [8], [9]], thus, affecting their quality and price. Green tea is not an exception.

Flavonoids, a class of plant secondary metabolites having a polyphenolic structure [10], are an important class of natural products widely found in flowers, fruits, vegetables, and certain beverages including tea [11]. The content of flavonoids in fresh tea leaves is generally between 18% and 36% (dry weight) [12]. They have miscellaneous favorable biochemical and antioxidant effects associated with various diseases such as cancer, Alzheimer’s disease (AD), atherosclerosis, and so on [[13], [14], [15], [16]]. Flavonoids can be subdivided into different subgroups depending on the carbon of the C ring to which the B ring is attached, the degree of unsaturation and oxidation of the C ring, and the structural features of the C ring [10] (Fig. S2), namely flavones, flavonols, flavanones, flavanonols, flavanols or catechins, anthocyanins, chalcones, and isoflavones. Existing studies presented that the ratios of epicatechin gallate (ECG)/epigallocatechin gallate (EGCG) and flavonol tri-glycosides/flavonol di-glycosides could be used as indicators for distinguishing the different varieties and cultivars of tea plants [17]; catechins, kaempferol, and quercetin derivatives were key metabolites responsible for cultivar discrimination of 14 Wuyi rock tea cultivars [18]; cultivars suitable for manufacturing green tea contained higher levels of flavonoid glycosides [19]; the intercultivar difference of flavonol glycosides in the same type of teas was greater than that of catechins [20]; significant (*p* < 0.05) differences of ECG, chlorogenic acid (CGA), and myricetin glycosides existed in ‘Wuyi Rougui’ and ‘Wuyi Shuixian’ [21]; non-galloylated catechins, hydroxycinnamic acids, flavonol glycosides, and gallotannins were marker compounds responsible for distinguishing short (1–5 years) and long (6–8 years) aging time of crude Pu-erh tea [22]. However, these studies are based on catechins as well as flavonols and their glycosides, wherein the coverage of flavonoid metabolites detection is limited, and the diversity of flavonoid metabolites in green tea is largely unknown. The effects of the geographic origin and the storage time on flavonoid metabolites of the same green tea remain unclear at the same time.

Revealing diversities and differences in flavonoid metabolic profiles of the same green tea, and identifying crucial compounds will be helpful to understand the quality difference of the same green tea due to the cultivar and the geographic origin under the premise of the consistent picking conditions, agronomic management, and processing technology, and also discover the change of green tea with the increase of storage time. Therefore, in this study, we used the same green tea (Longjing tea) from the same company to explore the effect of the cultivar, the geographic origin, and the storage time on the metabolic profiles of flavonoids. Then, we classified the differential flavonoid metabolites among the three comparisons, analyzed the regular patterns between them, and proposed potential flavonoid markers for the traceability of the cultivar, geographic origin, and storage time of Longjing tea. The results obtained from this study will shed light on a better understanding of the effects of the cultivar, the geographic origin, and the storage time on green tea metabolomic compounds and are expected to provide a theoretical reference and objective basis for the tea authenticity detection of famous green teas.

## 2 Materials and methods

### 2.1 Experimental materials

A reliable comparison among different tea samples can be made when variables are kept to a minimum [23,24], such as green tea samples for comparison in this study were obtained from the same company, Hangzhou Longguan Industrial Co., Ltd. (Hangzhou, China) and other external factors were relatively consistent, for example, the fresh tea leaves picked for these four Longjing tea samples were in the same grade and the agronomic management of the tea plants was consistent; meanwhile, the four Longjing tea samples underwent the same processing technology, that is, fresh leaves picking, cooling, fixing, and drying as the national standard [6] stipulated.

Longjing tea is one of the most famous green teas in China, and it is also a protected geographical indication product in China. In the national standard, the major aspects of Longjing tea authenticity are the cultivars and the geographic origin of Longjing tea; therefore, the most planted tea plant cultivar Longjing 43 and Longjing populations, as well as the main production area Xihu production area and Qiantang production area were chosen. Moreover, green tea is sought for freshness, and there is a huge gap between the prices of fresh Longjing tea and aged Longjing tea. Thus, four kinds of Longjing tea, whose differences lie in the cultivar, the geographic origin, and the production year were used in this study. The four kinds of Longjing tea samples were specifically named as follows: 22XH43, 22XHLS, 22QT43, and 21XH43, where the first two digits indicated the production year (2022 and 2021), the middle two digits indicated the geographic origin (Xihu production area and Qiantang production), and the last two digits indicated the cultivar (Longjing 43 and Longjing populations). Xihu production area is the Xihu district of Hangzhou (N 30°10′-30°16′, E 120°4′-120°10′) and Qiantang production area are districts of Hangzhou except Xihu district (illustrated in Fig. S1 in detail, and information of Hangzhou is N 29°11′-30°33′, E 118°21′-120°30′, subtropical monsoon climate, the annual average temperature of 17.8 °C, the annual precipitation of 1454 mm, and the annual sunshine hours of 1765 h). All Longjing tea samples were stored in a 4 °C refrigerator after the purchase.

### 2.2 Chemicals

The chemical reagents were chromatographic-grade. Methanol and acetonitrile were purchased from Merck (Darmstadt, Germany). Formic acid was purchased from Aladdin (Shanghai, China). The water used was doubly deionized with a Milli-Q water purification system (Millipore, Bedford, MA, USA).

### 2.3 Analysis of flavonoid metabolites present in longjing tea samples

#### 2.3.1 Sample preparation and extraction

Samples were freeze-dried under vacuum by a lyophilizer (Scientz-100 F), then ground (30 Hz, 1.5 min) to powder form by a grinder (MM 400, Retsch). 50 mg of each sample powder was weighed and 1200 μL of −20 °C pre-cooled 70% methanolic aqueous internal standard extract was added. Vortex once every 30 min for 30 s, for a total of 6 times. After centrifugation (rotation speed 12000 rpm, 3 min), the supernatant was aspirated, and the sample was filtered through a microporous membrane (0.22 μm pore size) and stored in the injection vial for ultra-performance liquid chromatograph-electro-spray ionization-tandem mass spectrometer (UPLC-ESI-MS/MS) analysis.

#### 2.3.2 UPLC conditions

UPLC-ESI-MS/MS combined with widely targeted metabolomics were used to investigate the flavonoid metabolites in these teas.

The analysis was conducted by MetWare (Wuhan, China). The sample extracts were analyzed using a UPLC-ESI-MS/MS system (UPLC, ExionLC™ AD; MS, Applied Biosystems 6500 Q TRAP). The analytical conditions were as follows, UPLC: column, Agilent SB-C18 (1.8 μm, 2.1 mm * 100 mm); The mobile phase consisted of solvent A, pure water with 0.1% formic acid, and solvent B, acetonitrile with 0.1% formic acid. Sample measurements were performed with a gradient program that employed the starting conditions of 95% A and 5% B. Within 9 min, a linear gradient to 5% A, 95% B was programmed, and a composition of 5% A, 95% B was kept for 1 min. Subsequently, a composition of 95% A and 5.0% B was adjusted within 1.1 min and kept for 2.9 min. The flow velocity was set as 0.35 mL per minute; The column oven was set to 40 °C; The injection volume was 2 μL. The effluent was alternatively connected to an ESI-triple quadrupole-linear ion trap (QTRAP)-MS.

#### 2.3.3 ESI-QTRAP-MS/MS

The analysis was conducted by MetWare (Wuhan, China). The ESI source operation parameters were as follows: source temperature 500 °C; ion spray voltage (IS) 5500 V (positive ion mode)/-4500 V (negative ion mode); ion source gas I (GSI), gas II(GSII), and curtain gas (CUR) were set at 50, 60, and 25 psi, respectively; the collision-activated dissociation (CAD) was high. The triple quadrupole (QQQ) scans were acquired as multiple reaction monitoring (MRM) experiments with collision gas (nitrogen) set to medium. Declustering potential (DP) and collision energy (CE) for individual MRM transitions were done with further DP and CE optimization. A specific set of MRM transitions were monitored for each period according to the metabolites eluted within this period.

### 2.4 Qualitative and quantitative analyses

Qualitative analysis involved identifying metabolites by matching the retention time, fragmentation patterns, and accurate *m*/*z* values to the standards in the self-constructed metabolite database (MetWare, Wuhan, China). On the basis of signal intensities of metabolites obtained from characteristic ions, quantitative analysis was carried out. Chromatographic peaks were integrated and calibrated using MultiaQuant software. The relative content of the corresponding substance was represented by the peak area of each chromatographic peak.

### 2.5 Quality control analysis of samples

Quality control samples were prepared from a mixture of sample extracts and were used to analyze the repeatability of the samples under the same treatment method. One quality control sample was inserted between 10 testing samples to monitor the reproducibility of the analysis process.

### 2.6 Statistical analysis

The coefficient of variation (CV) is the ratio of the original data's standard deviation to the original data's average, which reflects the degree of data dispersion degree. The empirical cumulative distribution function (ECDF) is used to analyze the frequency of the CV of substances less than the reference value. The higher the proportion of substances with a low CV value of the QC sample, the more stable the experimental data: the proportion of substances with a CV value of QC sample less than 0.5 is higher than 85%, indicating that the experimental data is stable, and the substance with CV value less than 0.3 is higher than 75%, indicating that the experimental data is very stable. The tests were repeated in triplicate, and the result of each test was expressed as the average of three replicates. The one-way analysis of variance (ANOVA) [25,26] test was conducted to assess statistically significant differences with 0.05. Statistical analyses were performed with Microsoft Office Excel 2021 (Microsoft, Washington State, USA).

#### 2.6.1 Principal component analysis

Unsupervised principal component analysis (PCA) was performed by statistics function prcomp within R (www.r-project.org). The data was unit variance scaled before unsupervised PCA.

#### 2.6.2 Hierarchical cluster analysis and pearson correlation coefficients

The hierarchical cluster analysis (HCA) results of samples and metabolites were presented as heatmaps with dendrograms, while Pearson correlation coefficients (PCC) between samples were calculated by the cor function in R and presented as only heatmaps. Both HCA and PCC were carried out by the R package Complex-Heatmap. For HCA, normalized signal intensities of metabolites (unit variance scaling) are visualized as a color spectrum.

#### 2.6.3 Differential metabolites selected

For two-group analysis, differential metabolites were determined by variable importance projection (VIP, VIP >1) and absolute Log_2_(Fold Change) (|Log_2_FC| ≥ 1.0). For multi-group analysis, differential metabolites were determined by VIP (VIP >1) and *p*-value (*p*-value <0.05, ANOVA). VIP values were extracted from the orthogonal partial least squares discrimination analysis (OPLS-DA) result, which also contains score plots and permutation plots and were generated using the R package MetaboAnalystR. The data was log transform (log_2_) and mean centering before OPLS-DA. To avoid overfitting, a permutation test (200 permutations) was performed.

#### 2.6.4 Kyoto Encyclopedia of Genes and Genomes annotation and enrichment analysis

Identified metabolites were annotated using the Kyoto Encyclopedia of Genes and Genomes (KEGG) Compound database (http://www.kegg.jp/kegg/compound/), annotated metabolites were then mapped to the KEGG Pathway database (http://www.kegg.jp/kegg/pathway.html). Pathways with significantly regulated metabolites mapped were then fed into metabolite sets enrichment analysis (MSEA), their significance was determined by the hypergeometric test’s *p*-values.

## 3 Results and discussion

### 3.1 Overall determination of flavonoid metabolites in lLongjing tea

A total of 483 flavonoid metabolites containing 10 subgroups were identified from the four kinds of Longjing tea based on the UPLC-MS/MS detection platform and the self-built database of MetWare. They were 20 chalcones, 47 flavanols or catechins, 158 flavones, 142 flavonols, 38 flavanones, 10 flavanonols, 2 anthocyanins, 16 proanthocyanins, 37 tannins, and 13 other flavonoids. Results demonstrated that flavones, flavonols, flavanols or catechins, and tannins were the dominant flavonoid metabolites in the four kinds of Longjing tea; the differences of the relative contents of 10 flavonoid subgroups were observed between the four kinds of Longjing tea, seen in Fig. 1 (A). Fig. 1 (B) demonstrated the differential flavonoid metabolites contents between the four kinds of Longjing tea (from left to right were 22XH43, 22XHLS, 22QT43, and 21XH43), where the dynamic changes of metabolites attributing to the cultivar, the geographic origin, and storage time were illustrated clearly.

**Fig. 1:**
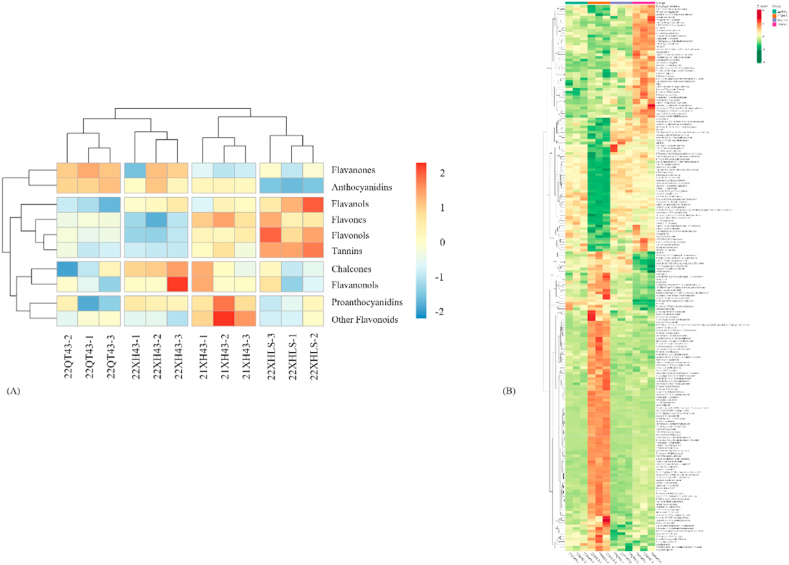
(A) Heat map of the differences in flavonoid subgroups between the four kinds of Longjing tea. (B) Heat map of differential metabolites in the four kinds of Longjing tea.

The result of cluster analysis in Fig. 1 (A) showed that flavonoid metabolites of group 22XH43 and group 22QT43 had a higher similarity, followed by group 22XH43 and group 21XH43 as well as group 22XH43 and group 22XHLS, which was consistent with the results of the PCA (Fig. 2.).

**Fig. 2:**
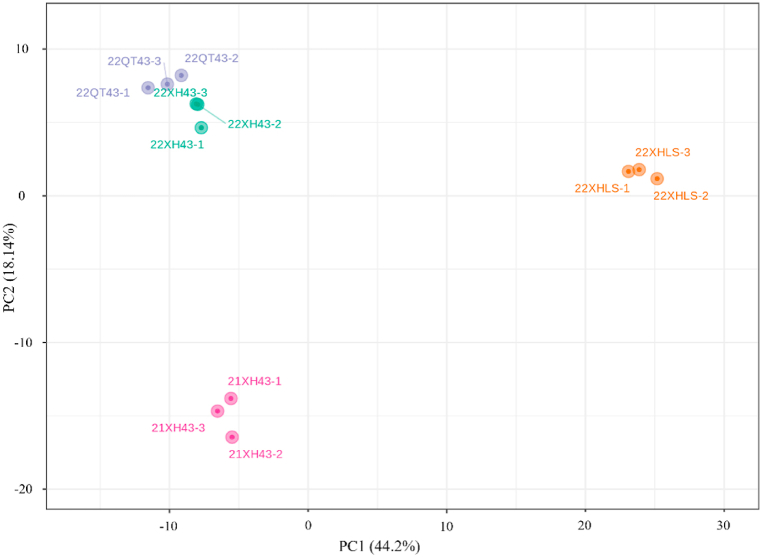
The PCA plot of flavonoids in the four kinds of Longjing tea.

In Fig. 2, the four kinds of Longjing tea were separated clearly, and the first two principal components, PC1, and PC2, represented a 44.2% and 18.14% contribution to the differences among kinds. To be more specific, the distance between group 22XH43 and group 22XHLS, as well as group 22XH43 and group 21XH43, appeared longer than the distance between group 22XH43 and group 22QT43 in Fig. 2, indicating that the differences in flavonoid metabolites between different cultivars of tea plants and the storage time of green tea were larger than those between the same green tea from different geographic origins.

### 3.2 Differential flavonoid metabolites in lLongjing tea

#### 3.2.1 Differential flavonoid metabolites between the cultivars

Determined by VIP >1 and |Log_2_FC| ≥ 1.0, there were 103 differential flavonoid metabolites of group 22XHLS relative to group 22XH43, including 7 flavonoid subgroups, which were chalcones, flavanols, flavones, flavonols, flavanones, tannins, and anthocyanidins, respectively, as detail numbers of each subgroup presented in Fig. 3 (A). In Fig. 3 (B), the volcano plots reflected the information on differential metabolite up-regulation and down-regulation, and their numbers were 83 up and 20 down, revealing the differences between transformation and generation of flavonoid compounds were the dominant reason for the differential flavonoid metabolites between different cultivars of tea plants.

**Fig. 3:**
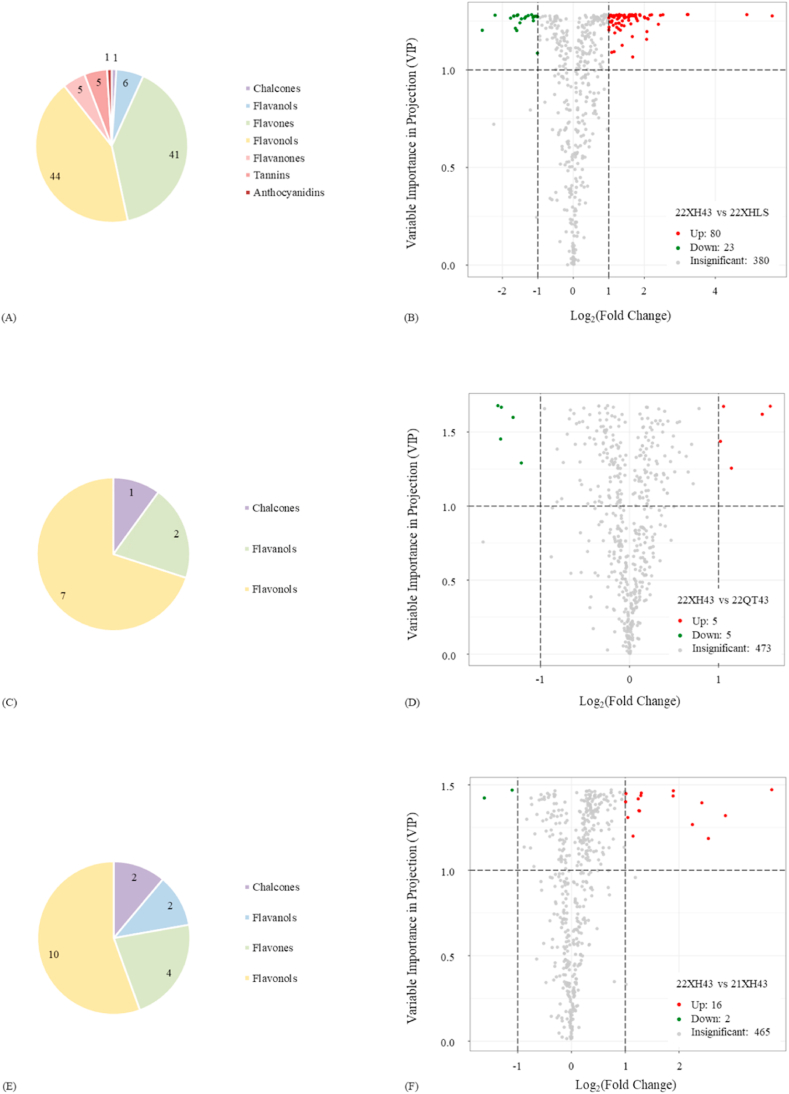
The analysis of differential flavonoid metabolites in the four kinds of Longjing tea. (A) The pie chart of flavonoid subgroups between group 22XH43 and group 22XHLS; (B) The volcano plot of group 22XH43 and group 22XHLS; (C) The pie chart of flavonoid subgroups between group 22XH43 and group 22QT43; (D) The volcano plot of group 22XH43 and group 22QT43; (E) The pie chart of flavonoid subgroups between group 22XH43 and group 21XH43; (F) The volcano plot of group 22XH43 and group 21XH43.

Specifically, differential flavonoid metabolites between group 22XH43 and group 21XH43 were screened (Fig. S3 (A)). Among 103 differential flavonoid metabolites, the sum of flavones and flavonols accounted more than 80% (85 of 103), and more than 90% (77 of 85) of them were the glycosides of flavones and flavonols. Besides, the number of glycosides in the other 5 subgroups of flavonoids also exceeded 50% (10 of 18), indicating that the difference in glycosides was the major difference between different cultivars of the same green tea with the same geographic origin and the same storage time. These findings were basically consistent with the existing studies, where flavonol glycosides were used as indicators for distinguishing the different cultivars of tea plants [17,18,21]. Whereas, the differential flavonoid metabolites with the largest |Log_2_FC| in this study were epigallocatechin3-*O*-(3-*O*-methyl)gallate, epicatechin-3-(3″-*O*-methyl)gallate, 5-hydroxy-6,7,3′,4′-tetramethoxyflavone and its isomer 5-hydroxy-3,7,3′,4′-tetramethoxyflavone (retusin), as well as syringetin and epigallocatechin 3,5-digallate, between the tea cultivar of Longjing 43 and the tea cultivar of Longjing populations, seen in Fig. S3 (B), suggesting the potentiality of other subgroups and compounds of flavonoid metabolites to be employed as the biochemical markers for tea cultivars traceability.

#### 3.2.2 Differential flavonoid metabolites between the geographic origins

Compared with the differential flavonoid metabolites between the cultivars, both the number and the subgroup of differential flavonoid metabolites between the geographic origins of Longjing tea had decreased, as Fig. 3 (C) illustrated. 10 differential flavonoid metabolites of group 22QT43 relative to group 22XH43 belonging to 3 subgroups of flavonoids were identified, with 5 metabolites up and 5 metabolites down (Fig. 3 (D)).

In this study, quercetin-3,4′-dimethyl ether, isorhamnetin-3-*O*-gallate, quercetin-3-*O*-(2″-*O*-rhamnosyl)rutinoside*, 4,4′-dihydroxy-2′-methoxychalcone (3-deoxysappanchalcone), and myricetin-3-*O*-(6″-galloyl)glucoside, were significantly upregulated, while kaempferol-3-*O*-neohesperidoside, epigallocatechin3-*O*-(3-*O*-methyl)gallate, epicatechin-3-(3″-*O*-methyl)gallate, kaempferol-3-*O*-(6″-galloyl)glucoside, and syringetin showed the opposite tendency (Fig. S4, compounds were sorted according to the value of Log_2_FC). Most differential flavonoid metabolites between group 22XH43 and group 22QT43 contained glycosylated structures or methylated structures, indicating that different plantation environments may act an important role in the structural modification of some flavonoid metabolites in Longjing tea with the same cultivar. The impact of the geographic origin on flavonoid metabolites was weaker than that of the cultivars, showing agreement with the existing study, in which authors held that the accumulation pattern of flavonoids was mainly dependent on the hereditary property of the tea plant [17]. Another reason may be that the Longjing tea from different geographic origins in this study was not far on the actual geographical distance, making them less different in the specific plantation environment. Despite this, as an agro-product, the geographic origin of tea is one of the most important attributes, sometimes it is regarded as the synonym of the tea quality [27]; therefore, the discovery and confirmation of the differential flavonoid metabolites of Longjing tea with the same cultivar but from different plantation environment was of great significance to actualize the geographic origin traceability of Longjing tea, and go further, in green tea.

#### 3.2.3 Differential flavonoid metabolites between the storage time

The quality of green tea is known to be deteriorated during storage [28,29], from the color, taste, the aroma, to the biological value. Existing researchers [30] discovered that the content of tea polyphenols and catechins decreased with the storage time and the ratio of ester catechins and non-ester catechins increased obviously. However, for flavonoid metabolites except catechins, no study has determined the changes in their contents under different storage times. This study compensated for this.

Shown in Fig. 3 (E), there were 18 differential flavonoid metabolites of group 21XH43 relative to group 22XH43 were identified, and they belonged to 4 subgroups of flavonoids. Among these 18 differential flavonoid metabolites, 16 compounds were of up-regulation and 2 compounds were of down-regulation (Fig. 3 (F)).

Kaempferol-3-*O*-rhamnoside (afzelin or kaempferin), quercetin-3-*O*-(2″-*O*-rhamnosyl)rutinoside*, quercetin-3-*O*-sambubioside-5-*O*-glucoside, quercetin-3-*O*-rutinoside-7-*O*-rhamnoside, and quercetin-3-*O*-α-l-rhamnopyranosyl-(1 → 3)-α-l-rhamnopyranosyl-(1 → 6)-β-d-glucopyranoside* were the top 5 up-regulated differential flavonoid metabolites between group 22XH43 and group 21XH43. Meanwhile, kaempferol-3-*O*-(2‴-*p*-coumaroyl)sophoroside-7-*O*-glucoside and phloretin were the 2 down-regulated differential flavonoid metabolites (Fig. S5, compounds were sorted according to the value of Log_2_FC). The diversities and differences in flavonoid metabolic profiles between groups 22XH43 and group 21XH43 were in the middle of the three comparisons in this study, which suggested that the influence of the hereditary property of the tea plant (different cultivars), storage time, and plantation environment (different geographic origins) on the accumulation pattern of flavonoids was decreasing successively.

### 3.3 KEGG enrichment analysis

Results of KEGG differential enrichment analysis were illustrated in Fig. S6. Obviously, the differentially significant metabolites annotated by KEGG, both in pathway and number, had the highest values in the comparison between the group 22XH43 and group 22XHLS, confirming the significant effect of the hereditary property on flavonoid metabolism in tea plants once more.

Biosynthesis of secondary metabolites (ko01110) as well as flavone and flavonol biosynthesis (ko00944) was the two pathways occurred all the three comparisons, followed by the flavonoid biosynthesis pathway (ko00941), which occurred in the two comparisons.

### 3.4 Potential flavonoid markers

In Fig. 4 (A), there were 5 common significant flavonoid metabolites between the comparison of cultivar (group 22XH43 and group 22XHLS) and the geographic origin (group 22XH43 and 22QT43), 4 common significant flavonoid metabolites between the cultivar (group 22XH43 and group 22XHLS) and the storage time (group 22XH43 and 21XH43), and 2 common significant flavonoid metabolites between the geographic origin (group 22XH43 and group 22QT43) and the storage time (group 22XH43 and 21XH43). 1 flavonoid metabolite was found significant in the all three comparisons, namely quercetin-3-*O*-(2″-*O*-rhamnosyl)rutinoside* (* represented the presence of indistinguishable isomers). Details of the total 108 differential flavonoid metabolites were given in Table 1. These significant differential flavonoid metabolites can be used as biochemical markers [5,31,32]. Therefore, on the basis of the above data and analysis, we’ve further classified the differential flavonoid metabolites among the three comparisons according to the subgroups of flavonoids, seen in Fig. 4 (B); a total of 108 significant differential flavonoid metabolites belonged to 8 flavonoid subgroups, with proanthocyanins and other flavonoids not included. Then, the regular patterns between these significant differential flavonoid metabolites were identified and classified into three subclasses, that is, glycosidified, methylated or methoxylated, and gallate (Table 1).

**Fig. 4:**
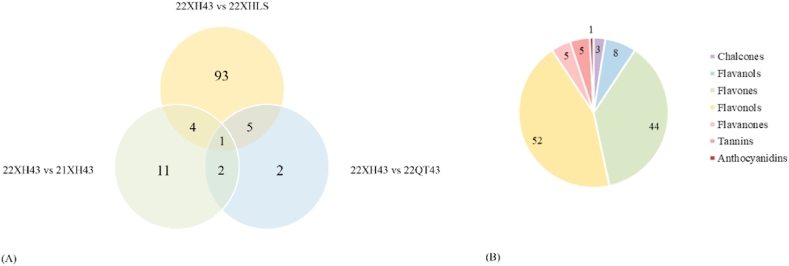
The analysis of significant flavonoid metabolites in the four kinds of Longjing tea. (A) Venn plot, numbers represent the significant metabolites; (B) The pie chart of flavonoid subgroups.

**Table 1:** Differential flavonoid metabolites of comparisons between cultivars, geographic origin, and storge time of Longjing tea.

Formula	Compounds	Level^a^	Cultivar	Geographic origin	Storage time	Class II^b^	Modified structures		
							Glycosidified	Methylated or methoxylated	Gallate
C_16_H_14_O_4_	(E)-Cardamonin	3	TRUE		TRUE	Chalcones		YES	
C_16_H_14_O_4_	3-Deoxysappanchalcone	3		TRUE		Chalcones		YES	
C_15_H_14_O_5_	Phloretin	1			TRUE	Chalcones			
C_21_H_24_O_11_	Catechin-5-*O*-glucoside	2	TRUE			Flavanols	YES		
C_29_H_22_O_14_	7,3′-Di-*O*-gallyoltricetiflavan	3	TRUE			Flavanols			YES
C_29_H_22_O_14_	7,4′-Di-*O*-galloyltricetiflavan	3	TRUE			Flavanols			YES
C_29_H_22_O_15_	Epigallocatechin-3,5-digallate	1	TRUE			Flavanols			YES
C_23_H_20_O_10_	Epicatechin-3-(3″-*O*-methyl)gallate	2	TRUE	TRUE		Flavanols		YES	YES
C_23_H_20_O_11_	Epigallocatechin3-*O*-(3-*O*-methyl)gallate	1	TRUE	TRUE		Flavanols		YES	YES
C_15_H_14_O_8_	3,3′,4′,5,6,7,8-Heptahydroxyflavan	2			TRUE	Flavanols			
C_24_H_22_O_11_	Epigallocatechin-3-*O*-(3,5-*O*-dimethyl)gallate	3			TRUE	Flavanols		YES	YES
C_21_H_20_O_10_	Apigenin-8-C-glucoside (Vitexin)	3	TRUE			Flavones	YES		
C_26_H_28_O_14_	Apigenin-6-C-(2″-xylosyl)glucoside	1	TRUE			Flavones	YES		
C_25_H_26_O_13_	Apigenin-6-C-xyloside-8-C-arabinoside	1	TRUE			Flavones	YES		
C_25_H_26_O_13_	Apigenin-6,8-di-C-arabinoside*	1	TRUE			Flavones	YES		
C_27_H_30_O_15_	Apigenin-6,8-di-C-glucoside (Vicenin)	1	TRUE			Flavones	YES		
C_25_H_26_O_13_	Apigenin-6-C-arabinoside-8-C-xyloside*	1	TRUE			Flavones	YES		
C_22_H_22_O_11_	Chrysoeriol-5-*O*-glucoside	3	TRUE			Flavones	YES	YES	
C_22_H_22_O_11_	Chrysoeriol-8-C-glucoside (Scoparin)	3	TRUE			Flavones	YES	YES	
C_22_H_22_O_11_	Diosmetin-6-C-glucoside	3	TRUE			Flavones	YES	YES	
C_22_H_22_O_11_	Hispidulin-8-C-glucoside	3	TRUE			Flavones	YES	YES	
C_26_H_28_O_14_	Hispidulin-8-C-(2″-*O*-xylosyl)xyloside	3	TRUE			Flavones	YES	YES	
C_28_H_32_O_16_	Hispidulin-8-C-(2″-*O*-glucosyl)glucoside	3	TRUE			Flavones	YES	YES	
C_26_H_28_O_14_	Isoschaftoside	1	TRUE			Flavones	YES		
C_26_H_28_O_14_	Isovitexin-8-*O*-xyloside	1	TRUE			Flavones	YES		
C_26_H_28_O_14_	Isovitexin-2″-*O*-xyloside	1	TRUE			Flavones	YES		
C_27_H_30_O_16_	Isoorientin-7-*O*-glucoside	2	TRUE			Flavones	YES		
C_27_H_30_O_15_	Isovitexin-7-*O*-glucoside (Saponarin)	2	TRUE			Flavones	YES		
C_27_H_30_O_15_	Isovitexin-4′-*O*-glucoside (Isosaponarin)	1	TRUE			Flavones	YES		
C_24_H_22_O_14_	Luteolin-7-*O*-(6″-malonyl)glucoside*	1	TRUE			Flavones	YES		
C_25_H_26_O_14_	Luteolin-6,8-di-C-arabinoside	3	TRUE			Flavones	YES		
C_27_H_30_O_16_	Luteolin-7-*O*-gentiobioside	2	TRUE			Flavones	YES		
C_27_H_30_O_15_	Luteolin-6-C-glucoside-7-*O*-rhamnoside	3	TRUE			Flavones	YES		
C_33_H_40_O_19_	Luteolin-7-*O*-rutinoside-5-*O*-rhamnoside	1	TRUE			Flavones	YES		
C_17_H_14_O_7_	Jaceosidin	2	TRUE			Flavones		YES	
C_23_H_24_O_12_	Jaceosidin-7-*O*-Glucoside*	1	TRUE			Flavones	YES	YES	
C_16_H_12_O_7_	Nepetin	1	TRUE			Flavones		YES	
C_22_H_22_O_12_	Nepetin-7-*O*-alloside*	1	TRUE			Flavones	YES	YES	
C_27_H_32_O_15_	Neoeriocitrin	3	TRUE			Flavones	YES		
C_26_H_28_O_15_	Orientin-6-C-arabinoside	3	TRUE			Flavones	YES		
C_26_H_28_O_15_	Orientin-7-*O*-arabinoside	3	TRUE			Flavones	YES		
C_19_H_18_O_7_	Retusin*	1	TRUE			Flavones		YES	
C_24_H_26_O_12_	Rhamnazine-5-*O*-glucoside	2	TRUE			Flavones	YES	YES	
C_24_H_26_O_12_	Rhamnazine-4′-*O*-glucoside	2	TRUE			Flavones	YES	YES	
C_17_H_14_O_7_	Tricin	1	TRUE			Flavones		YES	
C_23_H_24_O_12_	Tricin-7-*O*-glucoside*	1	TRUE			Flavones	YES	YES	
C_27_H_30_O_14_	Vitexin-2″-*O*-rhamnoside	1	TRUE			Flavones	YES		
C_27_H_30_O_15_	Vitexin-2″-*O*-galactoside	1	TRUE			Flavones	YES		
C_19_H_18_O_7_	5-Hydroxy-6,7,3′,4′-tetramethoxyflavone*	1	TRUE			Flavones		YES	
C_25_H_24_O_15_	3′-*O*-Methyltricetin-7-*O*-(6″-malonyl)glucoside*	3	TRUE			Flavones	YES	YES	
C_24_H_26_O_12_	3′,5′-Dihydroxy-5,7,4′-trimethoxyflavone glucoside	2	TRUE			Flavones	YES	YES	
C_27_H_30_O_16_	Orientin-7-*O*-glucoside	3	TRUE		TRUE	Flavones	YES		
C_17_H_14_O_6_	Dihydroxy-dimethoxyflavone	3			TRUE	Flavones		YES	
C_17_H_16_O_5_	7,4′-Dimethoxy-5-hydroxyflavone	3			TRUE	Flavones		YES	
C_34_H_42_O_21_	Chrysoeriol-6,8-di-C-glucoside-7-*O*-glucoside	3			TRUE	Flavones	YES		
C_21_H_20_O_10_	Kaempferol-7-*O*-rhamnoside	2	TRUE			Flavonols	YES		
C_23_H_22_O_12_	Kaempferol-3-*O*-(6″-*O*-acetyl)glucoside	1	TRUE			Flavonols	YES		
C_24_H_22_O_14_	Kaempferol-3-*O*-(6″-malonyl)galactoside*	1	TRUE			Flavonols	YES		
C_24_H_22_O_14_	Kaempferol-3-*O*-(6″-malonyl)glucoside*	1	TRUE			Flavonols	YES		
C_27_H_30_O_15_	Kaempferol-3-*O*-robinobioside (Biorobin)	3	TRUE			Flavonols	YES		
C_27_H_30_O_16_	Kaempferol-3,7-*O*-diglucoside	3	TRUE			Flavonols	YES		
C_28_H_24_O_15_	Kaempferol-3-*O*-(6″-galloyl)galactoside	1	TRUE			Flavonols	YES		
C_36_H_36_O_18_	Kaempferol-3-*O*-[2-*O*-(*trans*-*p*-coumaroyl)-3-*O*-ɑ-d-glucopyranosyl]-ɑ-d-glucopyranoside	2	TRUE			Flavonols	YES		
C_42_H_46_O_23_	Kaempferol-3-*p*-coumaroyldiglucoside-7-glucoside	2	TRUE			Flavonols	YES		
C_17_H_14_O_7_	Quercetin-3,3′-dimethyl ether	3	TRUE			Flavonols		YES	
C_21_H_20_O_12_	Quercetin-7-*O*-glucoside	1	TRUE			Flavonols	YES		
C_21_H_18_O_13_	Quercetin-4′-*O*-glucuronide	3	TRUE			Flavonols	YES		
C_23_H_22_O_13_	Quercetin-3-*O*-(6″-*O*-acetyl)galactoside	3	TRUE			Flavonols	YES		
C_23_H_20_O_14_	Quercetin-3-*O*-(2″-*O*-acetyl)glucuronide	3	TRUE			Flavonols	YES		
C_23_H_22_O_13_	Quercetin-3-*O*-(6″-*O*-acetyl)glucoside	1	TRUE			Flavonols	YES		
C_24_H_22_O_15_	Quercetin-7-*O*-(6″-malonyl)glucoside	1	TRUE			Flavonols	YES		
C_26_H_28_O_16_	Quercetin 3-*O*-glucoside-7-*O*-xyloside	3	TRUE			Flavonols	YES		
C_27_H_30_O_16_	Quercetin-3-*O*-robinobioside*	1	TRUE			Flavonols	YES		
C_27_H_30_O_16_	Quercetin-3-*O*-rutinoside (Rutin)	1	TRUE			Flavonols	YES		
C_27_H_30_O_17_	Quercetin-3,7-di-*O*-glucoside	2	TRUE			Flavonols	YES		
C_28_H_24_O_16_	Quercetin-3-*O*-(6″-*O*-galloyl)galactoside	1	TRUE			Flavonols	YES		
C_30_H_26_O_14_	Quercetin-3-*O*-(6″-*O*-*p*-coumaroyl)galactoside	2	TRUE			Flavonols	YES		
C_24_H_22_O_16_	Myricetin-3-*O*-(6″-malony)glucoside	3	TRUE			Flavonols	YES		
C_22_H_22_O_12_	Isorhamnetin-3-*O*-glucoside*	1	TRUE			Flavonols	YES	YES	
C_22_H_22_O_12_	Isorhamnetin-7-*O*-glucoside (Brassicin)*	1	TRUE			Flavonols	YES	YES	
C_28_H_32_O_16_	Isorhamnetin-3-*O*-neohesperidoside*	3	TRUE			Flavonols	YES	YES	
C_28_H_32_O_16_	Isorhamnetin-3-*O*-rutinoside (Narcissin)*	3	TRUE			Flavonols	YES	YES	
C_24_H_24_O_13_	Isorhamnetin-3-*O*-(6″-acetylglucoside)	3	TRUE			Flavonols	YES	YES	
C_25_H_24_O_15_	Isorhamnetin-3-*O*-(6″-malonyl)glucoside*	3	TRUE			Flavonols	YES	YES	
C_22_H_22_O_13_	Laricitrin-3-*O*-glucoside	1	TRUE			Flavonols	YES	YES	
C_28_H_32_O_16_	Rhamnetin-3-*O*-rutinoside	3	TRUE			Flavonols	YES	YES	
C_28_H_32_O_16_	Sexangularetin-3-*O*-glucoside-7-*O*-rhamnoside*	3	TRUE			Flavonols	YES	YES	
C_25_H_24_O_15_	Tamarixetin-3-*O*-(6″-malonyl)glucoside*	3	TRUE			Flavonols	YES	YES	
C_28_H_32_O_16_	Tamarixetin-3-*O*-rutinoside	3	TRUE			Flavonols	YES	YES	
C_28_H_32_O_16_	Tamarixetin-3-*O*-glucoside-7-*O*-rhamnoside*	3	TRUE			Flavonols	YES	YES	
C_17_H_14_O_7_	3,7-Di-*O*-methylquercetin	3	TRUE			Flavonols		YES	
C_28_H_32_O_16_	6-C-Methylquercetin-3-*O*-rutinoside	3	TRUE			Flavonols	YES	YES	
C_28_H_32_O_16_	3′-Methoxyquercetin-3-*O*-l-rhamnosyl (1 → 2)-glucopyranoside*	3	TRUE			Flavonols	YES	YES	
C_28_H_24_O_15_	Kaempferol-3-*O*-(6″-galloyl)glucoside	2	TRUE	TRUE		Flavonols	YES		
C_28_H_24_O_17_	Myricetin-3-*O*-(6″-galloyl)glucoside	3	TRUE	TRUE		Flavonols	YES		
C_17_H_14_O_8_	Syringetin	3	TRUE	TRUE		Flavonols		YES	
C_21_H_20_O_10_	Kaempferol-3-*O*-rhamnoside	2	TRUE		TRUE	Flavonols	YES		
C_33_H_40_O_20_	Quercetin 3-*O*-α-l-rhamnopyranosyl-(1 → 3) -α-l-rhamnopyranosyl-(1 → 6)-β-d-glucopyranoside*	1	TRUE		TRUE	Flavonols	YES		
C_27_H_30_O_15_	Kaempferol-3-*O*-neohesperidoside	1		TRUE		Flavonols	YES		
C_23_H_16_O_11_	Isorhamnetin-3-*O*-gallate	1		TRUE	TRUE	Flavonols		YES	YES
C_17_H_14_O_7_	Quercetin-3,4′-dimethyl ether	2		TRUE	TRUE	Flavonols		YES	
C_16_H_12_O_6_	Kaempferide*	3			TRUE	Flavonols		YES	
C_42_H_46_O_23_	Kaempferol-3-*O*-(2‴-*p*-coumaroyl)sophoroside-7-*O*-glucoside	3			TRUE	Flavonols	YES		
C_21_H_20_O_11_	Quercetin-3-*O*-rhamnoside (Quercitrin)	3			TRUE	Flavonols	YES		
C_32_H_38_O_21_	Quercetin-3-*O*-sambubioside-5-*O*-glucoside	3			TRUE	Flavonols	YES		
C_33_H_40_O_20_	Quercetin-3-*O*-rutinoside-7-*O*-rhamnoside	1			TRUE	Flavonols	YES		
C_33_H_40_O_20_	Quercetin-3-*O*-(2″-*O*-rhamnosyl)rutinoside*	1	TRUE	TRUE	TRUE	Flavonols	YES		
C_27_H_32_O_15_	Eriodictyol-7-*O*-rutinoside (Eriocitrin)	3	TRUE			Flavanones	YES		
C_23_H_26_O_11_	Hesperetin-3′-*O*-glucuronide	3	TRUE			Flavanones	YES	YES	
C_28_H_34_O_15_	Hesperetin-7-*O*-neohesperidoside (Neohesperidin)*	2	TRUE			Flavanones	YES	YES	
C_28_H_34_O_15_	Hesperetin-7-*O*-rutinoside (Hesperidin)*	3	TRUE			Flavanones	YES	YES	
C_29_H_36_O_15_	Methylhesperidin	3	TRUE			Flavanones	YES	YES	
C_15_H_8_O_8_	3-*O*-Methylellagic acid	1	TRUE			Tannin		YES	
C_17_H_12_O_8_	3,3′,4-*O*-Trimethylellagic acid	2	TRUE			Tannin		YES	
C_20_H_16_O_13_	Ellagic acid-4-*O*-glucoside	1	TRUE			Tannin	YES		
C_20_H_16_O_12_	Ellagic acid-4-*O*-rhamnoside	3	TRUE			Tannin	YES		
C_21_H_18_O_13_	3′-*O*-Methylellagic acid 4-*O*-glucoside	1	TRUE			Tannin	YES	YES	
C_21_H_21_O_12_+	Delphinidin-3-*O*-glucoside (Mirtillin)	2	TRUE			Anthocyanidins	YES		
Total	118		103	10	18		93	52	7

Notes: ^a^ Metabolites identification level, 1: The matching scores of secondary mass spectrometry and retention time (RT) of metabolites to database standards were greater than 0.7; 2: The matching scores of secondary mass spectrometry and RT of metabolites to database standards were 0.5–0.7; 3: The RT, precursor ion (Q1), product ion (Q3), declustering potential (DP), and collision energy (CE) of metabolites were consistent with the database standards. ^b^ Secondary classification of metabolites.

#### 3.4.1 Glycosidified

Glycosidification had the largest proportion in the structural modification classification of significant differential flavonoid metabolites, where flavonol O- glycosides (FOG) and flavone C-glycosides (FCG) occupied the majority and flavone O-glycosides appeared at the same time. It seemed difficult to produce consistent regularity for the glycosidification of flavones and flavonols, because of the diversity of their aglycones, glycosyl groups, and the attached sites. For a specific flavone or flavonol, for instance, kaempferol, 13 kaempferol O-glycosides were identified in the comparisons of cultivar, the geographic origin, and the storage time, containing mono- and di-glycosides, as well as acetyl, malonyl, galloyl, and coumaroyl glycosides. Determine the total amount of flavones or flavonols after glycoside hydrolysis [33] and calculate the ratio [17] of different glycosides for the specific flavones or flavonols are two methods that can be tried. However, the problem of the former is that the change trend of the glycosides of the same flavones or flavonols in the same comparison is different. Taking this study for example, kaempferol-7-*O*-rhamnoside and kaempferol-3-*O*-rhamnoside were both significant differential flavonoid metabolites of cultivars, however, kaempferol-7-*O*-rhamnoside was relatively high in group 22XH43, and kaempferol-3-*O*-rhamnoside was relatively high in group 22XHLS (Fig. S1 (A)); the simple hydrolysis will offset the difference between them. The problem of the latter is that FOG and FCG usually have isomers, which are difficult to distinguish, meanwhile, the lack of standards makes their research accessibility decrease. Significant differential flavonoid metabolites in Table 1 with level of 1, having the relative high concentration, standards, and without indistinguishable isomers may be potential flavonoid markers, such as rutin (quercetin-3-*O*-rutinoside), vitexin-2″-*O*-rhamnoside, and isosaponarin (isovitexin-4′-*O*-glucoside) in the cultivar, and kaempferol-3-*O*-neohesperidoside in the geographic origin in this study; the combination of several significant differential flavonoid metabolites and the joint distinguish method worth trial at the same time.

#### 3.4.2 Methylated or methoxylated

The proportion of methylation or methoxylation in significant differential flavonoid metabolites was in the middle level, whereas, their differences were extremely significant, such as epigallocatechin3-*O*-(3-*O*-methyl)gallate, epicatechin-3-(3″-*O*-methyl)gallate, methylhesperidin, 6-C-methylquercetin-3-*O*-rutinoside, and syringetin in the cultivar comparison (Fig. S3 (B)), quercetin-3,4′-dimethyl ether and syringetin in the geographic origin comparison (Fig. S4.), all were the top 20% differential metabolites.

Other classes of compounds with methylation or methoxylation modification structures requiring attention were chrysoeriol (3′-*O*-methylluteolin), diosmetin (5,7,3′-trihydroxy-4′-methoxyflavone), hispidulin (6-methoxy-5,7,4′-trihydroxyflavone), jaceosidin (4′,5,7-trihydroxy-3′,6-dimethoxyflavone), nepetin (6-methoxyluteolin), retusin (quercetin- 3, 3′, 4′, 7- tetramethylether), tricin (3′,5′-dimethoxyflavone), isorhamnetin (3′-methylquercetin), laricitrin (3′-*O*-methylmyricetin), rhamnetin (7-*O*-methylquercetin), rhamnazin (3′,7-di-*o*-methylquercetin), sexangularetin (8-methoxykaempferol), tamarixetin (4′-*O*-methylquercetin), syringetin (3′,5′-*O*-dimethylmyricetin), hesperetin (3′,5,7-trihydroxy-4′-methoxyflavanone), and their glycosides. Their methoxylation modifications occurred on the aglycone, and subsequently, glycosidification continued on the basis of the methoxylated compound or not.

It should be noted that the structural modifications of glycosidification and methylation or methoxylation to existing flavonoids sometimes do not exist alone, where 32 significant differential flavonoid metabolites both contained glycosidified and methylated or methoxylated structures identified in the total 118 significant differential flavonoid metabolites. This phenomenon not only indicates that the structural modification is a major reason for the differences of flavonoids metabolites in cultivars, geographic origins, and storage time in Longjing tea but also brings challenges to the determination of the specific significant differential flavonoid metabolites, since the more modified structures, the more complex the molecular structure, the greater the possibility of isomers, which means more precise separation and determination methods required.

#### 3.4.3 Gallate

In tea, gallic acid is usually combined with catechin to form catechin gallate, which is also the case in this study. Besides, they were important in distinguishing the cultivar and the geographic origin of Longjing tea, such as epigallocatechin3-*O*-(3-*O*-methyl)gallate and epicatechin-3-(3″-*O*-methyl)gallate, which were both significant differential metabolites between the comparison of different cultivars and geographic origins (Fig. S3 (B) and Fig. S4), though, both of them were also modified by methylation. In addition, gallic acid is also combined with other flavonoids, and one of them was isorhamnetin in this study (Table 1), to form isorhamnetin-3-*O*-gallate. In this study, isorhamnetin-3-*O*-gallate acted as an indicator in identifying Longjing tea with different geographic origins and different storage time.

#### 3.4.4 Chalcones

Chalcones were another class of meaningful compounds identified in this study with the flavonoid marker potentiality. Although among the significant differential flavonoid metabolites of cultivars, geographic origins, and storage time, only 3 compounds belonged to chalcones, whereas, they were distributed in all three comparisons, especially (E)-cardamonin, which was a significant differential flavonoid metabolite in cultivar and storage time of Longjing tea in this study, indicating that chalcones as flavonoid markers have valuable application prospects in the identification of Longjing tea cultivars, geographic origins, and storage time. Phloretin, a dihydrochalcone, which can be found in apple tree leaves [34] and flowers [35], is the only compound among the 118 significant differential metabolites that is not modified by glycosidification, methylation or methoxylation, and gallic acid. In this study, the content of phloretin in Longjing tea decreased significantly with the increase of storage time, seen Fig. S5. Notwithstanding, there is a difficulty with chalcones, that is, their relative contents are not high.

## 4 Conclusion

In this study, a total of 483 flavonoid metabolites with 10 subgroups included were identified and 118 of them were differential flavonoid metabolites. Glycosidification accounted for the largest proportion of structural modifications of differential flavonoid metabolites, followed by methylation or methoxylation, and gallic acid structures also existed in a small number of differential flavonoid metabolites. Chalcones were another class of flavonoids that have the potential to be flavonoid markers. This study has enriched the understanding of the effects of the cultivar, the geographic origin, and the storage time on the flavonoid metabolic profiles of Longjing tea, and provided potential flavonoid markers for the traceability of green tea in different cultivars, geographic origins, and storage time. Further studies are needed to explore the flavonoid metabolic profiles of other green teas and identify flavonoid markers with greater universality based on the more comprehensive and in-depth analysis of green tea flavonoid metabolic profiles; the effects of agronomic managements on the flavonoid metabolic profiles of Longjing tea and other green teas with the same cultivar and the geographic origin are also worth study.

## Author contribution statement

**Xiao-Lan Yu:** Conceived and designed the experiments; Performed the experiments; Analyzed and interpreted the data; Wrote the paper.

**Jia Li, Yanqing Yang, Jiayi Zhu:** Analyzed and interpreted the data.

**Haibo Yuan, Yongwen Jiang:** Contributed reagents, materials, analysis tools or data.

## Data availability statement

Data will be made available on request.

## Declaration of competing interest

The authors declare that they have no known competing financial interests or personal relationships that could have appeared to influence the work reported in this paper.

## Figures and Tables

**figs1:**
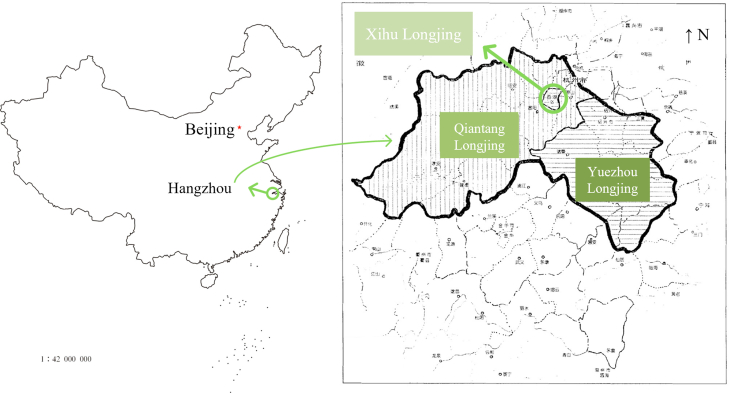


**figs2:**
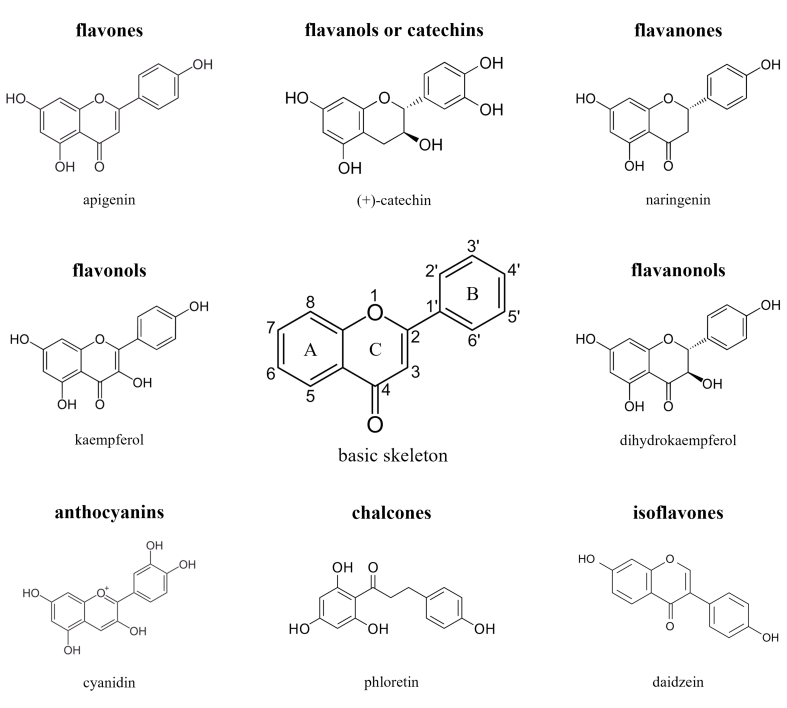


**figs3:**
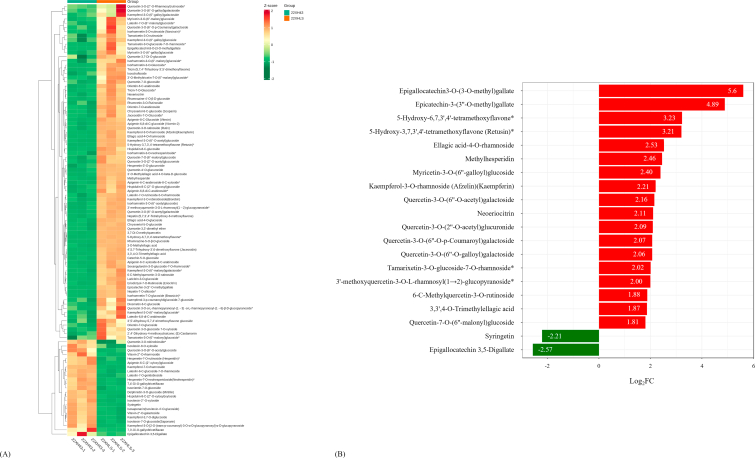


**figs4:**
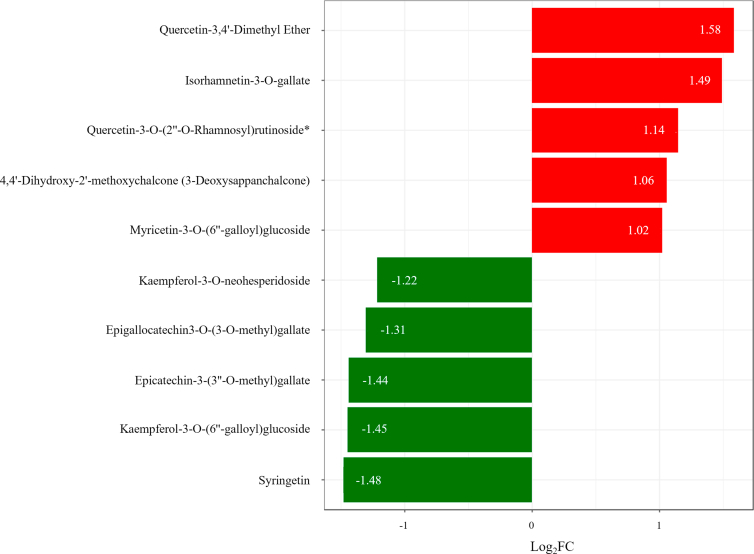


**figs5:**
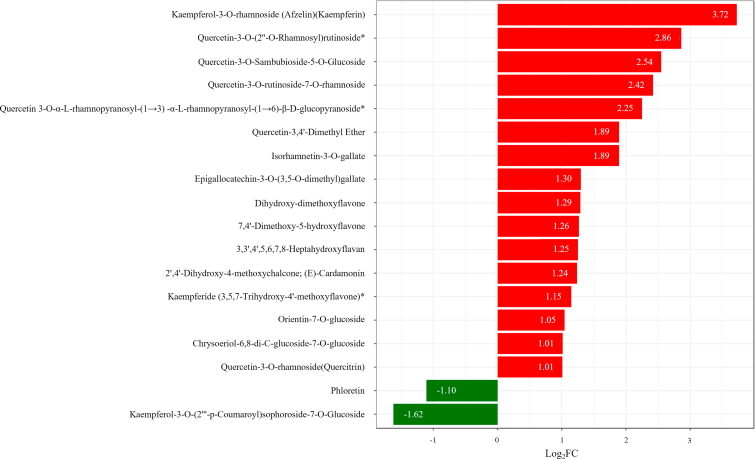


**figs6:**
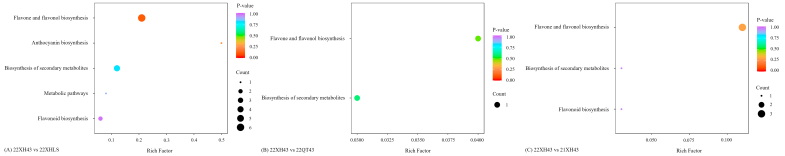


## References

[1] Han Z.-X. (2016). Green tea flavour determinants and their changes over manufacturing processes. Food Chem..

[2] Tan H.R. (2019). Characterisation of key odourants in Japanese green tea using gas chromatography-olfactometry and gas chromatography-mass spectrometry. LWT--Food Sci. Technol..

[3] Han Z. (2022). LC-MS based metabolomics and sensory evaluation reveal the critical compounds of different grades of Huangshan Maofeng green tea. Food Chem..

[4] Xing L., Zhang H., Qi R., Tsao R., Mine Y. (2019). Recent advances in the understanding of the health benefits and molecular mechanisms associated with green tea polyphenols. J. Agric. Food Chem..

[5] Engelhardt U.H. (2020). Tea chemistry – what do and what don't we know? – a micro review. Food Res. Int..

[6] GB/T 18650-2008 (2008).

[7] Pop R.M. (2013). UHPLC/PDA-ESI/MS analysis of the main berry and leaf flavonol glycosides from different Carpathian Hippophaë rhamnoides L. Varieties. Phytochem. Anal..

[8] Shen Y. (2018). Establishment of a rapid method to quantify eight flavonol glycosides for quality assessment of Red Toon using UPLC. Acta Chromatogr..

[9] Cuoco G., Mathe C., Vieillescazes C. (2014). Liquid chromatographic analysis of flavonol compounds in green fruits of three Rhamnus species used in Stil de grain. Microchem. J..

[10] Panche A.N., Diwan A.D., Chandra S.R. (2016). Flavonoids: an overview. J. Nutr. Sci..

[11] Martínez-Damián M.T., Mejía-Muñoz J.M., Colinas-León M.T., Hernández-Epigmenio F., Cruz-Alvarez O. (2021). Nutritional value, bioactive compounds and capacity antioxidant in edible flowers of dahlia. Acta Sci. Pol. Hortorum Cultus.

[12] Wan X.-C. (2011).

[13] Sun M. (2022). Potential therapeutic use of plant flavonoids in AD and PD. Heliyon.

[14] Uddin M.S. (2020). Molecular insight into the therapeutic promise of flavonoids against Alzheimer's disease. Molecules.

[15] Salvamani S., Gunasekaran B., Shaharuddin N.A., Ahmad S.A., Shukor M.Y. (2014). Antiartherosclerotic effects of plant flavonoids. BioMed Res. Int..

[16] Abotaleb M. (2019). Flavonoids in cancer and apoptosis. Cancers.

[17] Fang Z.-T. (2021). Accumulation pattern of catechins and flavonol glycosides in different varieties and cultivars of tea plant in China. J. Food Compos. Anal..

[18] Chen S. (2018). Metabolite profiling of 14 Wuyi Rock tea cultivars using UPLC-QTOF MS and UPLC-QqQ MS combined with chemometrics. Molecules.

[19] Li P. (2018). Metabolomic analysis reveals the composition differences in 13 Chinese tea cultivars of different manufacturing suitabilities. J. Sci. Food Agric..

[20] Zheng X.-Q. (2018). Screening the cultivar and processing factors based on the flavonoid profiles of dry teas using principal component analysis. J. Food Compos. Anal..

[21] Zhao F. (2014). Simultaneous determination of caffeine and some selected polyphenols in Wuyi Rock tea by high-performance liquid chromatography. J. Agric. Food Chem..

[22] Ren Y. (2022). Metabolomics, sensory evaluation, and enzymatic hydrolysis reveal the effect of storage on the critical astringency-active components of crude Pu-erh tea. J. Food Compos. Anal..

[23] Carloni P. (2013). Antioxidant activity of white, green and black tea obtained from the same tea cultivar. Food Res. Int..

[24] Shi Y. (2022). Comprehensive investigation on non-volatile and volatile metabolites in four types of green teas obtained from the same tea cultivar of Longjing 43 (Camellia sinensis var. sinensis) using the widely targeted metabolomics. Food Chem..

[25] Stahle L., Wold S. (1989). Analysis of variance (ANOVA). Chemometr. Intell. Lab. Syst..

[26] Gill G.S., Kumar A., Agarwal R. (2013). Nondestructive grading of black tea based on physical parameters by texture analysis. Biosyst. Eng..

[27] Wadood S.A., Boli G., Xiaowen Z., Hussain I., Yimin W. (2020). Recent development in the application of analytical techniques for the traceability and authenticity of food of plant origin. Microchem. J..

[28] Ananingsih V.K., Sharma A., Zhou W. (2013). Green tea catechins during food processing and storage: a review on stability and detection. Food Res. Int..

[29] Mirasoli M. (2014). Electronic nose and chiral-capillary electrophoresis in evaluation of the quality changes in commercial green tea leaves during a long-term storage. Talanta.

[30] Su X. (2020). Effects of environmental factors on storage quality of flat green tea and preliminary study on shelf life. Sci. Technol. Food Ind..

[31] Gulati A. (2009). Catechin and catechin fractions as biochemical markers to study the diversity of Indian tea (Camellia sinensis (L.) O. Kuntze) germplasm. Chem. Biodivers..

[32] Fraser K. (2013). Analysis of metabolic markers of tea origin by UHPLC and high resolution mass spectrometry. Food Res. Int..

[33] Wang H., Wen M.-C., Jiang Z.-D., Zha M.-Y., Zhang L. (2021). Optimization of the acid hydrolysis conditions of flavonoid glycosides in different teas and determination for the content of 3 kinds of flavonols before and after acid hydrolysis. J. Food Saf. Qual..

[34] Zhao Y. (2013). Study on the dynamic changes of phlorizin and phloretin in leave and branches of apple tree. Food Res. Dev..

[35] Yin Y., Li Y., Han H., Gong X., Zeng C. (2016). HPLC determination of phloretin in Pumila Flos. Cent. South Pharm..

